# Long‐read sequencing reveals SVA insertion in *AP3B1* causing Hermansky–Pudlak syndrome 2

**DOI:** 10.1111/pai.70406

**Published:** 2026-07-10

**Authors:** Naomi Baba, Ahmed Elashy, Oliver Wegehaupt, Barbara Zieger, Melisa Felek, Micol Ferro, Kimberly Gilmour, Ute Fischer, Arndt Borkhardt, Stephan Ehl, David Koppstein, Sujal Ghosh

**Affiliations:** ^1^ Department of Pediatric Oncology, Hematology and Clinical Immunology, Medical Faculty Heinrich‐Heine‐University, University Hospital Düsseldorf Düsseldorf Germany; ^2^ Cancer Bioinformatics and Multiomics (ED08) German Cancer Research Center Heidelberg and German Cancer Consortium (DKTK), Partner Site Essen/Düsseldorf Düsseldorf Germany; ^3^ Center for Digital Medicine (ZDM), Faculty of Medicine University Hospital Düsseldorf Düsseldorf Germany; ^4^ Institute for Immunodeficiency, Center for Chronic Immunodeficiency, Medical Center, Faculty of Medicine University of Freiburg Freiburg Germany; ^5^ Department of Pediatrics and Adolescent Medicine, Division of Pediatric Hematology and Oncology, Medical Center, Faculty of Medicine University of Freiburg Freiburg Germany; ^6^ Department of Immunology Camelia Botnar Laboratories, Great Ormond Street Hospital (GOSH) London UK

**Keywords:** AP3B1, Hermansky–Pudlak syndrome type 2, inborn errors of immunity, long‐read sequencing, lysosomal trafficking disorders, retrotransposon insertion

To the Editor,

Hermansky‐Pudlak syndrome type 2 (HPS2) is an autosomal recessive inborn of immunity (IEI) caused by biallelic variants in *AP3B1*. It is characterized by oculocutaneous albinism, bleeding diathesis, and congenital neutropenia. Immunologic abnormalities include defects in T and NK cell cytotoxicity, with some patients developing hemophagocytic lymphohistiocytosis.^1^, ^2^, ^3^, ^4^ *AP3B1* encodes the β3A subunit of adaptor protein complex 3 (AP‐3), which is essential for lysosomal protein sorting and vesicle formation.

Although whole‐exome sequencing (WES) is widely used in the diagnosis of IEIs, many patients remain genetically unresolved. Here, we describe a patient with a clinical phenotype consistent with HPS2 where repeated WES identified only a single pathogenic allele in *AP3B1*. Through targeted long‐read sequencing and transcript analysis, we identified the second mutation and established the molecular diagnosis. These findings extend the mutational spectrum of *AP3B1* and illustrate the utility of long‐read sequencing in resolving genetically unexplained cases.

Informed consent was obtained from the family. NK‐cell degranulation was assessed by CD107a upregulation, NK cell cytotoxicity by a chromium‐release assay, and platelet degranulation by CD63 and CD62 upregulation.^2^ Whole‐exome sequencing was performed using Illumina platforms, and variants were analyzed according to ACMG/ACGS guidelines. Targeted long‐read sequencing of the AP3B1 locus was performed using adaptive sampling with Readfish on an Oxford Nanopore platform. RNA was isolated from whole blood, and 3′ rapid amplification of cDNA ends (3´‐RACE) was performed using gene‐specific primers to assess transcript structure. RNA sequencing libraries were prepared using standard Illumina protocols. Long‐read and RNA‐seq data were analyzed using established pipelines.^5^, ^6^, ^7^, ^8^, ^9^

Our female patient is the first child of healthy, unrelated Ukrainian parents. Nystagmus was noticed at 8 weeks of age. Ophthalmologic evaluation showed pendular nystagmus, convergent strabismus, foveal hypoplasia, and reduced visual acuity. She was later diagnosed with oculocutaneous albinism. Psychomotor development was delayed; she achieved head control at 10 months, sat independently at 11 months, and walked at 18 months. Muscular hypertonia was noted. Mild dysmorphic features were observed including posteriorly rotated ears and a flat nasal bridge, while growth parameters remained normal (Figure 1A). Although not considered classic manifestations of HPS2, mild developmental and dysmorphic features have occasionally been reported in AP3B1 deficiency. Consistent with HPS2, she had light blond hair and skin hypopigmentation. Microscopic examination of the hair shaft revealed reduced pigmentation and clumping (Figure 1A,B).

**FIGURE 1:**
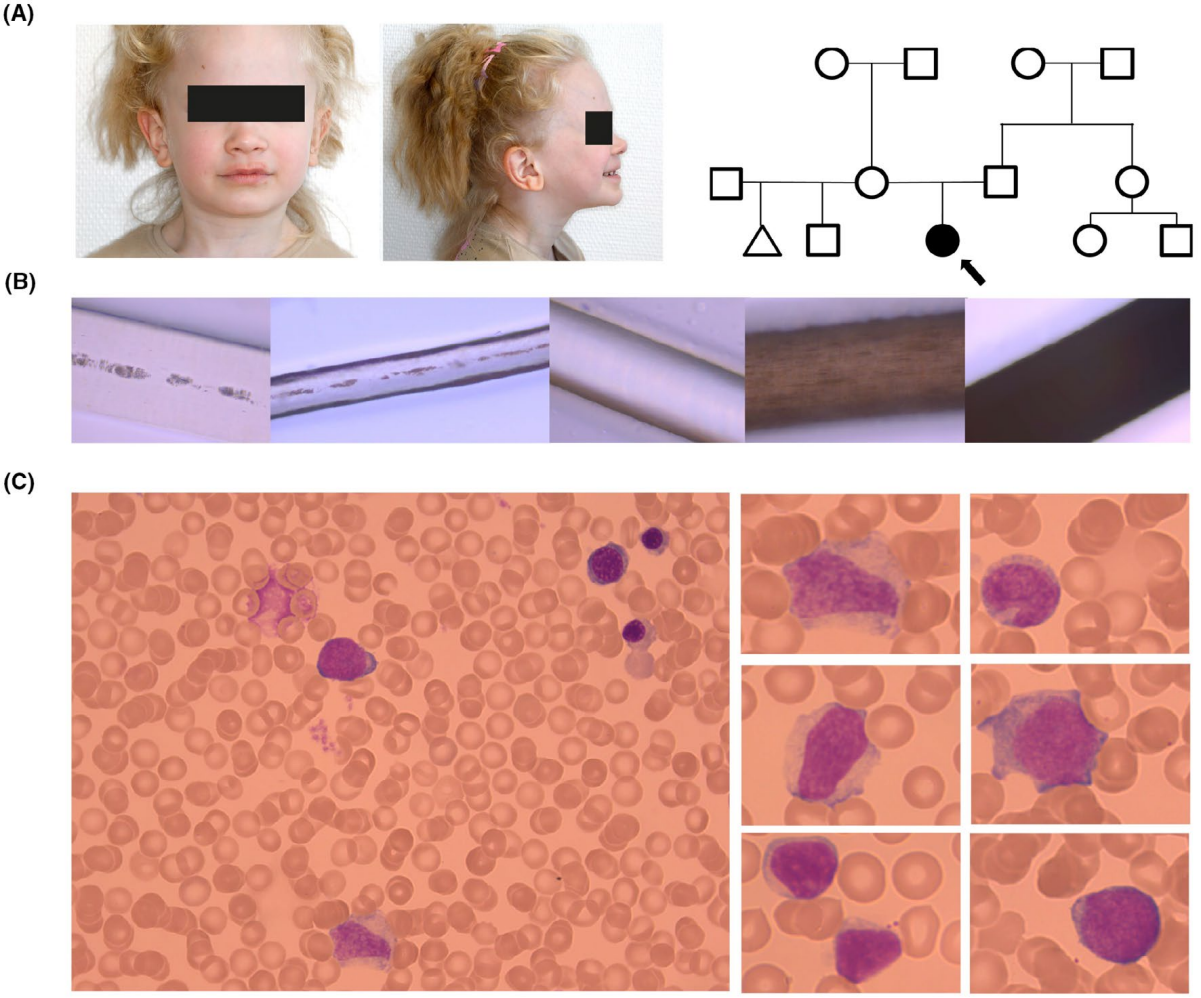
Clinical phenotype, hair shaft abnormalities, and hematologic features (A) Representative clinical images demonstrating oculocutaneous hypopigmentation with light hair and skin. The patient shows mild craniofacial dysmorphism, including low‐set, posteriorly rotated ears and a flat nasal bridge. The family pedigree is shown; the affected individual is indicated by a filled symbol, and parents are unaffected heterozygous carriers. (B) Light microscopy of hair shafts demonstrating marked hypopigmentation with uneven melanin distribution and characteristic pigment clumping in patient hair (left and middle). Hair from an unrelated blond control (center) shows no visible pigmentation. Hair from the father (second from right) demonstrates dense pigmentation. Hair from the mother (right) is artificially colored and not evaluable for endogenous pigmentation; her natural hair color is dark. (C) Bone marrow aspirate demonstrates marked granulocytic hypoplasia with scarcity of mature neutrophils and accumulation of early myeloid precursors showing hypogranular cytology consistent with a granule‐biogenesis defect. Erythropoiesis is relatively preserved with normoblastic maturation, while megakaryocytes are rare in the examined fields. No increase in blasts, ring sideroblasts, or hemophagocytic activity is observed.

She was hospitalized at 11 months of age with febrile neutropenia and developed recurrent infections. ANCs fluctuated between complete absence and 360/μl. Bone marrow biopsy demonstrated marked granulocytic hypoplasia with scarcity of mature neutrophils and accumulation of early myeloid precursors (Figure 1C). G‐CSF treatment initiated at 2 years improved neutrophil counts and prevented further severe infections. After dental surgery, she had one episode of prolonged postoperative bleeding without a history of recurrent epistaxis.

Immunological assessment revealed low NK cells which showed almost no CD107a upregulation upon stimulation, suggesting impaired degranulation capacity (Figure 2A). Chromium release assay suggested a complete defect in NK cell cytotoxicity (Figure 2B). Platelet degranulation assays demonstrated impaired CD63 upregulation upon thrombin stimulation, whereas CD62 expression increased normally, indicating preserved α‐granule release and impairment of lysosome‐related organelle exocytosis (Figure 2C). Transmission electron microscopy of platelets showed a marked reduction of small dense granules compared with controls (Figure 2D).

**FIGURE 2:**
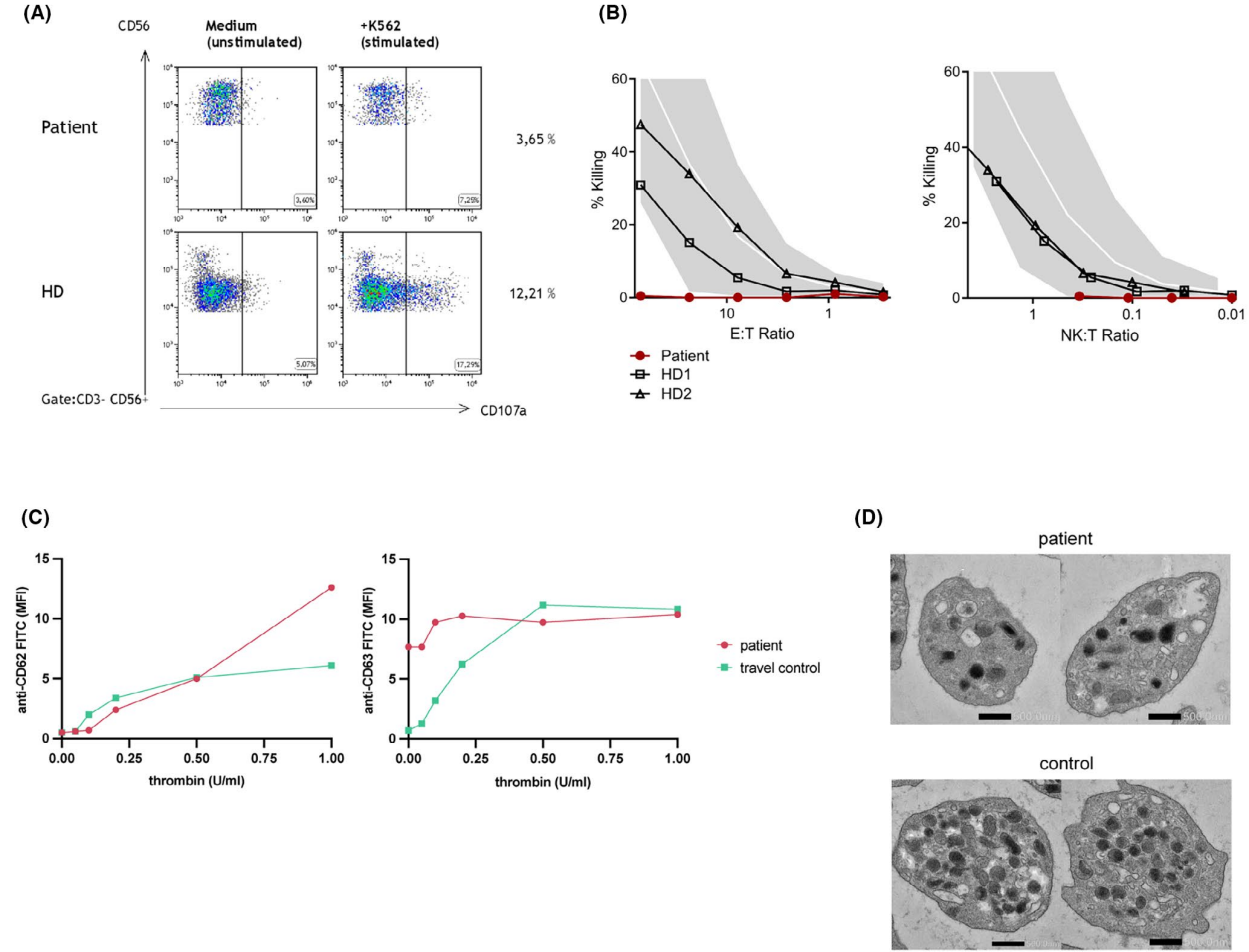
Functional and ultrastructural analysis of NK cells and platelet granules (A) NK‐cell degranulation was measured by CD107a surface expression on NK cells for both the medium and K562‐stimulated conditions. The healthy control (HD) shows an increase in CD107a expression after K562 stimulation (normal threshold >10%). The patient's NK cells display almost no increase in CD107a surface expression compared with the medium control, resulting in a ΔCD107a of <5% (B) NK‐cell cytotoxicity was measured by a 51Cr‐release assay using patients' PBMCs incubated with K562 target cells for 4 h. The effector‐to‐target (E:T) ratio included all PBMCs. NK‐to‐target (NK:T) ratio was determined by flow‐cytometric quantification of NK cells within PBMCs. Spontaneous release was <10%. The percentage of target cells killed are shown in red (patient) and black (healthy donor controls (HD1, HD2)). The gray area represents the mean (white line) ±2 SD of cytotoxicity from 31 non‐sequenced healthy controls. (C) Flow cytometric analysis of platelet activation following stimulation with increasing concentrations of thrombin. Surface expression of the lysosomal marker CD63 and the α‐granule marker CD62 is shown as median fluorescence intensity (MFI) for the patient (red circles) and a travel control (green squares). CD63 expression is increased at baseline in the patient but shows only limited up‐regulation upon thrombin stimulation, whereas CD62 up‐regulation is comparable between patient and control. (D) Representative transmission electron microscopy images of resting platelets from the patient (left) and healthy control (right). Control platelets contain numerous small, round, highly electron‐dense granules with homogeneous internal density, consistent with normal platelet dense granules. In contrast, platelets from the patient show a marked reduction of such highly electron‐dense granules across multiple examined platelets. Instead, the cytoplasm is dominated by larger, less electron‐dense granules with heterogeneous appearance, compatible with α‐granules, as well as vacuolar profiles. The overall granule distribution appears altered, with an absence of the characteristic dense granule population observed in control platelets. Scale bars, 500 nm.

Repeated WES performed at external diagnostic laboratories identified a heterozygous AP3B1 exon 8–11 deletion by NGS‐based CNV calling, which encodes the N‐terminal adaptin region essential for AP‐3 assembly.^1^, ^2^ However, no pathogenic second variant was detected. Subsequent long read sequencing with adaptive sampling targeting the *AP3B1* locus followed by Sniffles2 annotation revealed the precise 9.7 kb deletion (GRCh38 chr5:78,175,255_78,184,983del; NM_003664.5:c.787‐3320_1167 + 370del) supported by 8 breakpoint‐spanning reads. Additionally, this analysis revealed an additional heterozygous ~2.63 kb insertion in intron 7 supported by four high‐quality breakpoint‐spanning reads (GRCh38 chr5:78,205,013; NM_003664.5(AP3B1):c.786 + 11042_786 + 11043insSVA; Figure 3). Both variants were marked as PRECISE by Sniffles2. WhatsHap revealed that the insertion lay on a different haplotype phase than the exonic deletion. Alignment of the sequence with BLASTn^10^ returned 98% sequence identity to a SINE‐VNTR‐Alu (SVA) retrotransposon.

**FIGURE 3:**
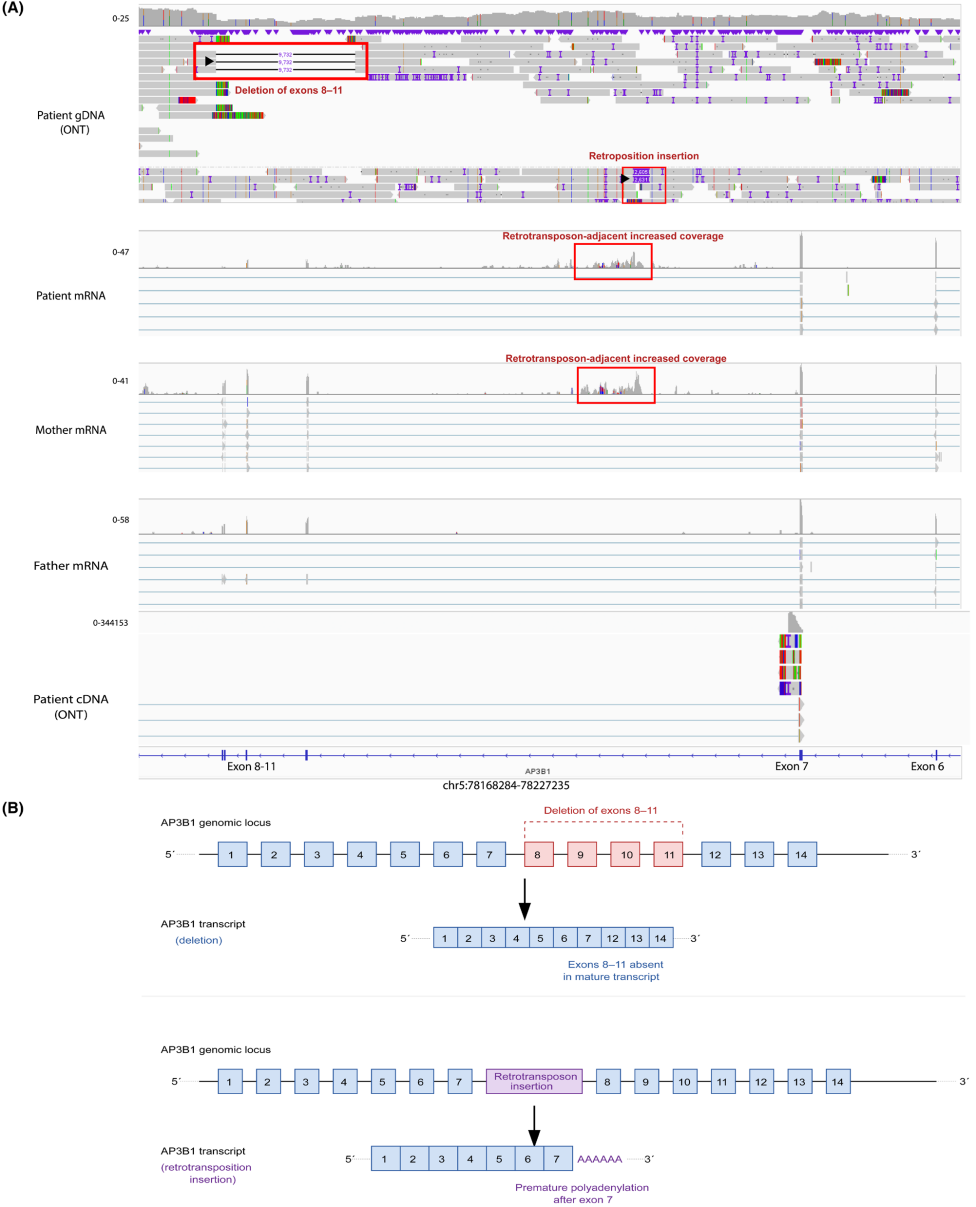
Integrated genomic and transcriptomic analysis of the *AP3B1* locus. (A) Genome tracks of the *AP3B1* locus. The upper track represents patient genomic DNA (gDNA) generated using long‐read sequencing with Readfish adaptive sampling. Red boxes highlight the exon 8–11 deletion and the retrotransposon insertion in intron 7. The subsequent tracks show patient, father, and mother mRNA sequencing data. An additional red box in the RNA‐seq coverage track indicates the marked increase in read depth across the deleted exonic region adjacent to the retrotransposon insertion. The patient cDNA track displays the 3′ RACE product sequenced by long‐read sequencing with representative reads demonstrating exon 8–11 deletion and premature polyadenylation in intron 7. (B) Model of the two *AP3B1* alleles and their inferred transcriptional products.

For segregation analysis, we performed mRNA sequencing (mRNA‐seq) in the patient and both parents. In the father, reads either included exons 8–11 of *AP3B1* (39.1%–48.2%) or skipped these exons (46.1%–52.6%) (Figure 3A; Figure S2), supporting heterozygosity for the transcript alteration. By contrast, all maternal reads retained exons 8–11. In the patient, some reads contained at least two of exons 8–11 (19.5%–30.9%), but these reads were not linked to upstream exons (Table S1). Both the mother's and child's mRNA displayed aberrant coverage adjacent to the retrotransposon insertion in intron 7 of *AP3B1* consistent with maternal inheritance and aberrant RNA processing at this locus (Figure 3A). To determine the effects of the retrotransposon, we performed 3´‐RACE on patient RNA using nested forward primers in exons 5 and 7 of *AP3B1* followed by long‐read sequencing. In contrast to mRNA‐seq, there was almost no coverage downstream of exon 7. Instead, reads terminating prematurely shortly after exon 7 downstream of a poly(A)_12_ stretch represented the dominant transcript (>340 k reads, Figure 3, Figure S1). This isoform is predicted to code for an additional 25 amino acids reading into intron 7 and is terminated by a stop codon 759 nucleotides prior to the poly(A) tail. In summary, we infer the presence of two *AP3B1* representative transcripts: the first has a loss of exons 8–11 from one copy of chromosome 5, and the second terminates via premature polyadenylation shortly after exon 7 (Figure 3B).

Transcriptome analysis showed separation between patient and controls by PCA (Figure S3), and gene set enrichment analysis revealed upregulation of Myc‐ and E2F‐related programs (Figure S4). Additionally, neutrophil granule and activation genes were upregulated, consistent with G‐CSF treatment. Interestingly, *AP3B1* itself was not dysregulated (Figure S5), suggesting that altered transcript structures, not altered gene expression, were responsible for the clinical phenotype. No transcriptional signature of lysosomal or vesicle trafficking pathways was detected, consistent with the posttranscriptional role of the AP‐3 complex.

HPS2 is characterized by defects in the AP‐3 complex. The clinical findings in our patient, including congenital neutropenia, impaired NK‐cell degranulation, abnormal platelet dense‐granule secretion, and oculocutaneous albinism, are consistent with AP‐3 deficiency. The molecular findings expand the mutational spectrum of AP3B1 and reveal a previously unrecognized disease mechanism mediated by a pathogenic SVA retrotransposon insertion. We identified two novel compound heterozygous variants in AP3B1: a deletion of exons 8–11 on the paternal allele and a previously unreported ~2.63 kb SVA insertion in intron 7 on the maternal allele.

Targeted long‐read sequencing using adaptive sampling was essential to resolve and phase the intronic retrotransposon relative to the exonic deletion. SVA retrotransposons are active mobile elements in the human genome and can disrupt gene function through aberrant splicing or transcriptional dysregulation. Although not previously reported in HPS, intronic SVA insertions are a recognized disease mechanism, for example in X‐linked dystonia‐parkinsonism caused by aberrant splicing of TAF1.^11^, ^12^ In our patient, SVA insertion activates a cryptic polyadenylation site causing premature termination of transcription, which has been described in L1 but not SVA retrotransposons.^13^ The RNA‐seq coverage downstream of the retrotransposon insertion in the mother and child (Figure 3A) correlates with the presence of SVA and could reflect a degradation product. Further investigation into the consequences of premature transcript termination is warranted.

Although short‐read WES remains the current diagnostic standard, it systematically fails to detect intronic variants and mobile elements.^8^ In contrast, long‐read sequencing enables contiguous, haplotype‐resolved reads that facilitate the detection of structural variants including retrotransposon insertions. In conclusion, we report a case of HPS2 caused by an exonic deletion and a pathogenic intronic SVA insertion in *AP3B1*, representing a novel molecular mechanism in HPS2. As sequencing technologies evolve, long‐read methods will play a central role in defining the genomic landscape of IEIs and other diseases.

## AUTHOR CONTRIBUTIONS

**Oliver Wegehaupt:** Investigation; writing – review and editing; formal analysis; visualization. **Stephan Ehl:** Formal analysis; writing – review and editing; resources. **Micol Ferro:** Investigation; writing – review and editing. **Barbara Zieger:** Writing – review and editing; investigation; resources; formal analysis; visualization. **Kimberly Gilmour:** Writing – review and editing; resources. **Melisa Felek:** Writing – review and editing; investigation. **Ahmed Elashy:** Data curation; formal analysis; visualization; writing – original draft; investigation. **Arndt Borkhardt:** Writing – review and editing; resources; formal analysis. **Ute Fischer:** Writing – review and editing; resources. **Sujal Ghosh:** Conceptualization; investigation; funding acquisition; writing – original draft; writing – review and editing; formal analysis; project administration; supervision; resources; visualization. **David Koppstein:** Conceptualization; supervision; investigation; visualization; formal analysis; writing – original draft; writing – review and editing; project administration; funding acquisition; resources. **Naomi Baba:** Investigation; writing – original draft; visualization; formal analysis; data curation.

## FUNDING INFORMATION

NB and SG received funding from the Elterninitiative Kinderkrebsklinik e.V. DK and UF received funding from the Bundesministerium für Forschung, Technologie und Raumfahrt (BMFTR) grant number 01KD2410A, EDI‐4‐ALL. SE received funding from the DFG SFB1160 (256073931).

## CONFLICT OF INTEREST STATEMENT

The authors declare no conflicts of interest.

## Supporting information


**Figure S1.** Zoom‐in of the patient long‐read sequencing cDNA gene products at the premature polyadenylation site adjacent to a cryptic poly(A) stretch. AP3B1, right to left.
**Figure S2:** Percentage of reads supporting the wild type gene structure containing exons 8–11 at the *AP3B1* locus.
**Figure S3:** Principal component analysis of RNA‐seq datasets for the family and a healthy control.
**Figure S4:** Gene set enrichment analysis performed using FGSEA on the patient vs. healthy control RNA‐seq datasets.
**Figure S5:** Violin plots of AP3B1 expression from RNA‐seq. (B) Exon‐level counts per million of *AP3B1* derived from RNA‐seq.
**Table S1:** mRNA‐seq read counts covering the *AP3B1* locus exons 8–11. A, aberrant, missing these exons; I, uninformative; W, wild type (containing at least two of exons 8–11).

## Data Availability

The data that support the findings of this study are available from the corresponding author upon reasonable request.
