# Supplementary material for: Long‐read sequencing reveals SVA insertion in AP3B1 causing Hermansky–Pudlak syndrome 2
Source: Pediatr Allergy Immunol. 2026 Jul 10;37(7):e70406. doi: 10.1111/pai.70406 (PMC13354856; doi:10.1111/pai.70406)
Supplement: Supplementary file 1 — Figure S1. Zoom‐in of the patient long‐read sequencing cDNA gene products at the premature polyadenylation site adjacent to a cryptic poly(A) stretch. AP3B1, right to left. Figure S2: Percentage of reads supporting the wild type gene structure containing exons 8–11 at the AP3B1 locus. Figure S3: Principal component analysis of RNA‐seq datasets for the family and a healthy control. Figure S4: Gene set enrichment analysis performed using FGSEA on the patient vs. healthy control RNA‐seq datasets. Figure S5: Violin plots of AP3B1 expression from RNA‐seq. (B) Exon‐level counts per million of AP3B1 derived from RNA‐seq. Table S1: mRNA‐seq read counts covering the AP3B1 locus exons 8–11. A, aberrant, missing these exons; I, uninformative; W, wild type (containing at least two of exons 8–11). [file PAI-37-e70406-s001.docx]

**Figure S1**


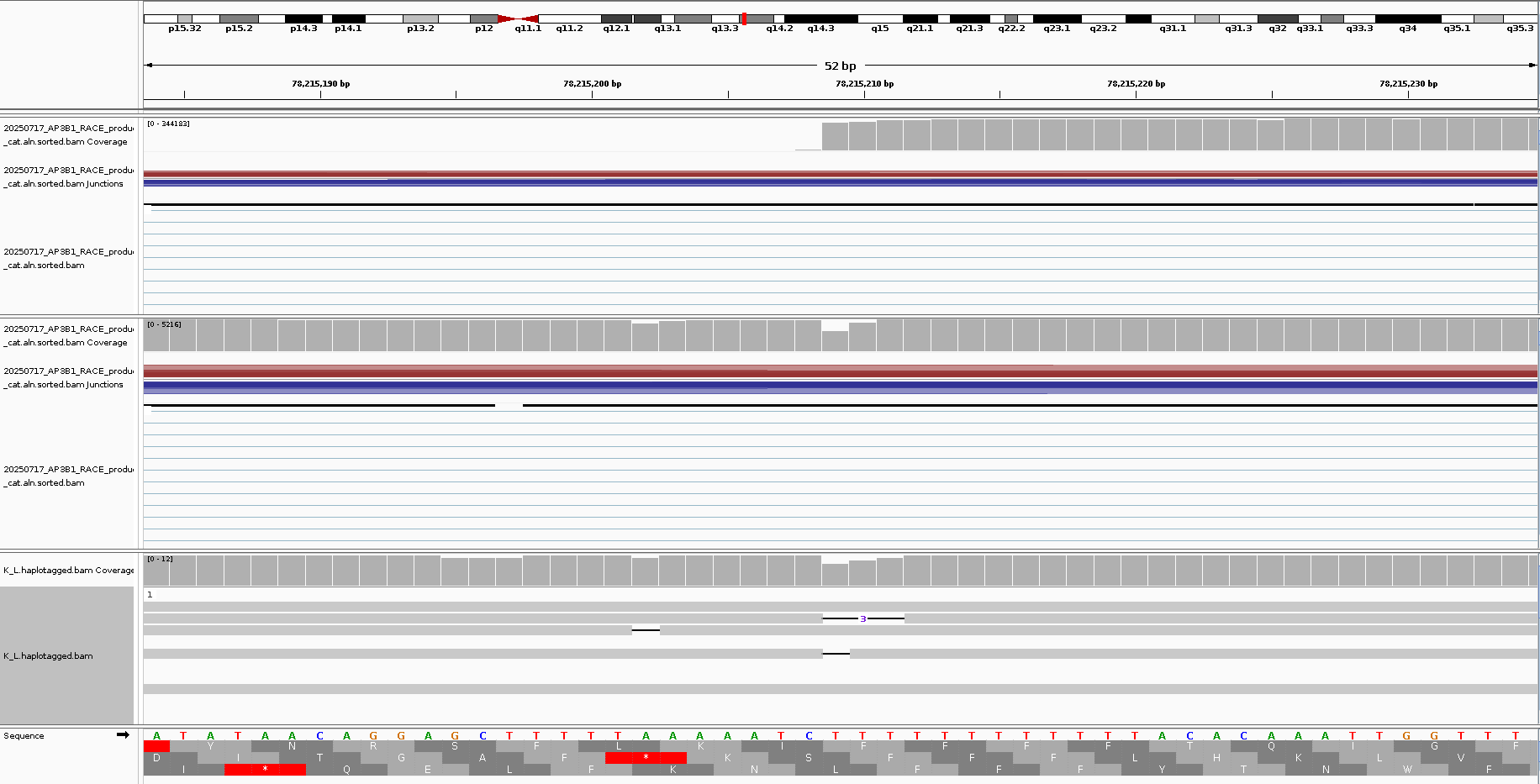


Zoom-in of the patient long-read sequencing cDNA gene products at the premature polyadenylation site adjacent to a cryptic poly(A) stretch. AP3B1, right to left.

**Figure S2**


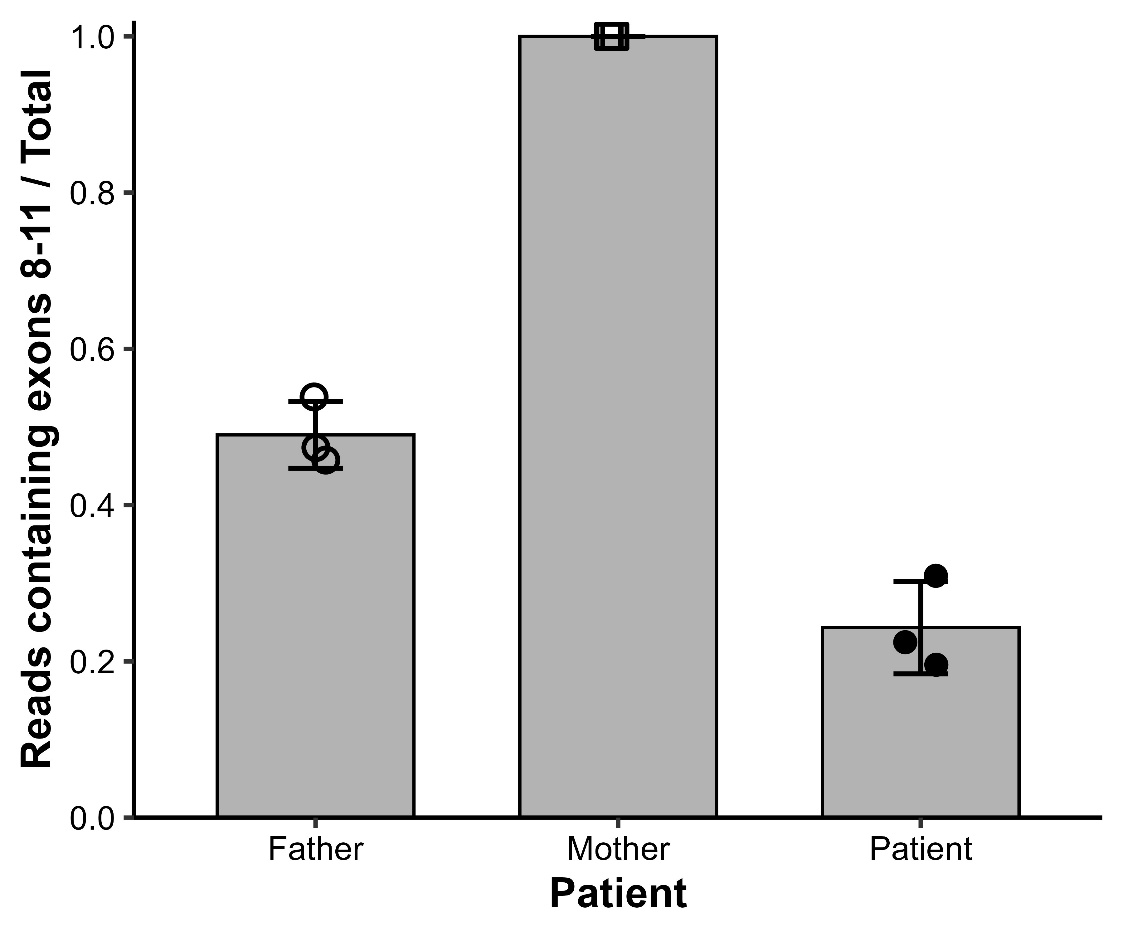


Percentage of reads supporting the wild type gene structure containing exons 8-11 at the *AP3B1* locus.

**Figure S3**

**
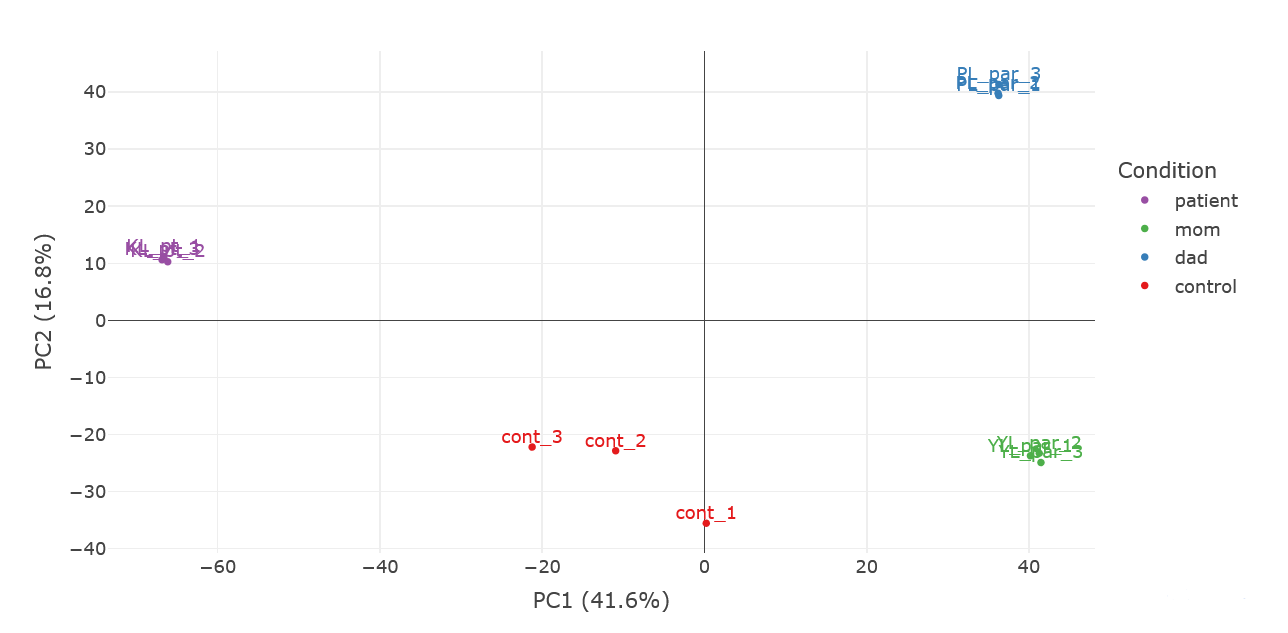
**

Principal component analysis of RNA-seq datasets for the family and a healthy control.

**Figure S4**

**
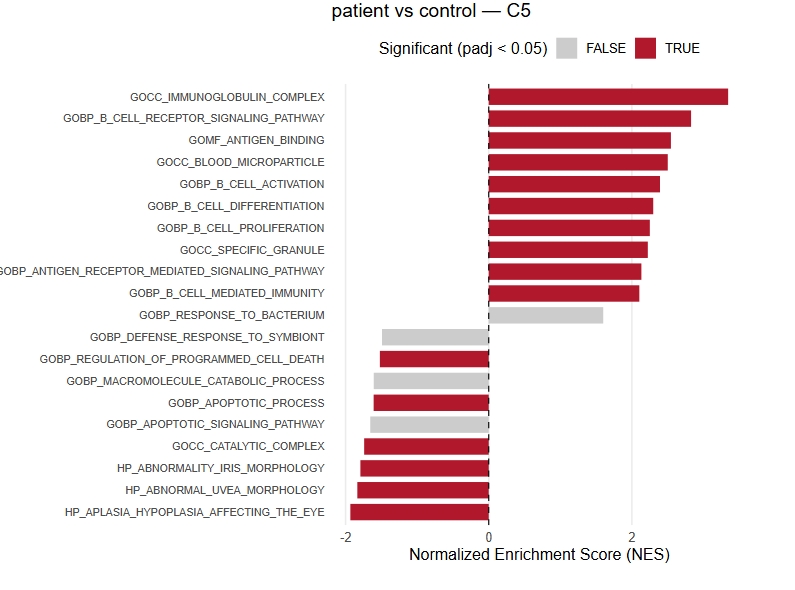
**

**
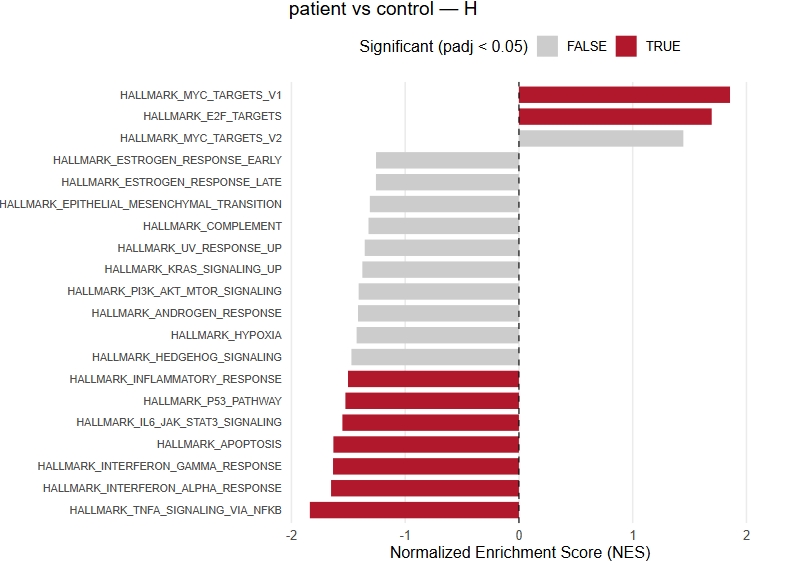
**

Gene set enrichment analysis performed using FGSEA on the patient vs. healthy control RNA-seq datasets.

**Figure S5**
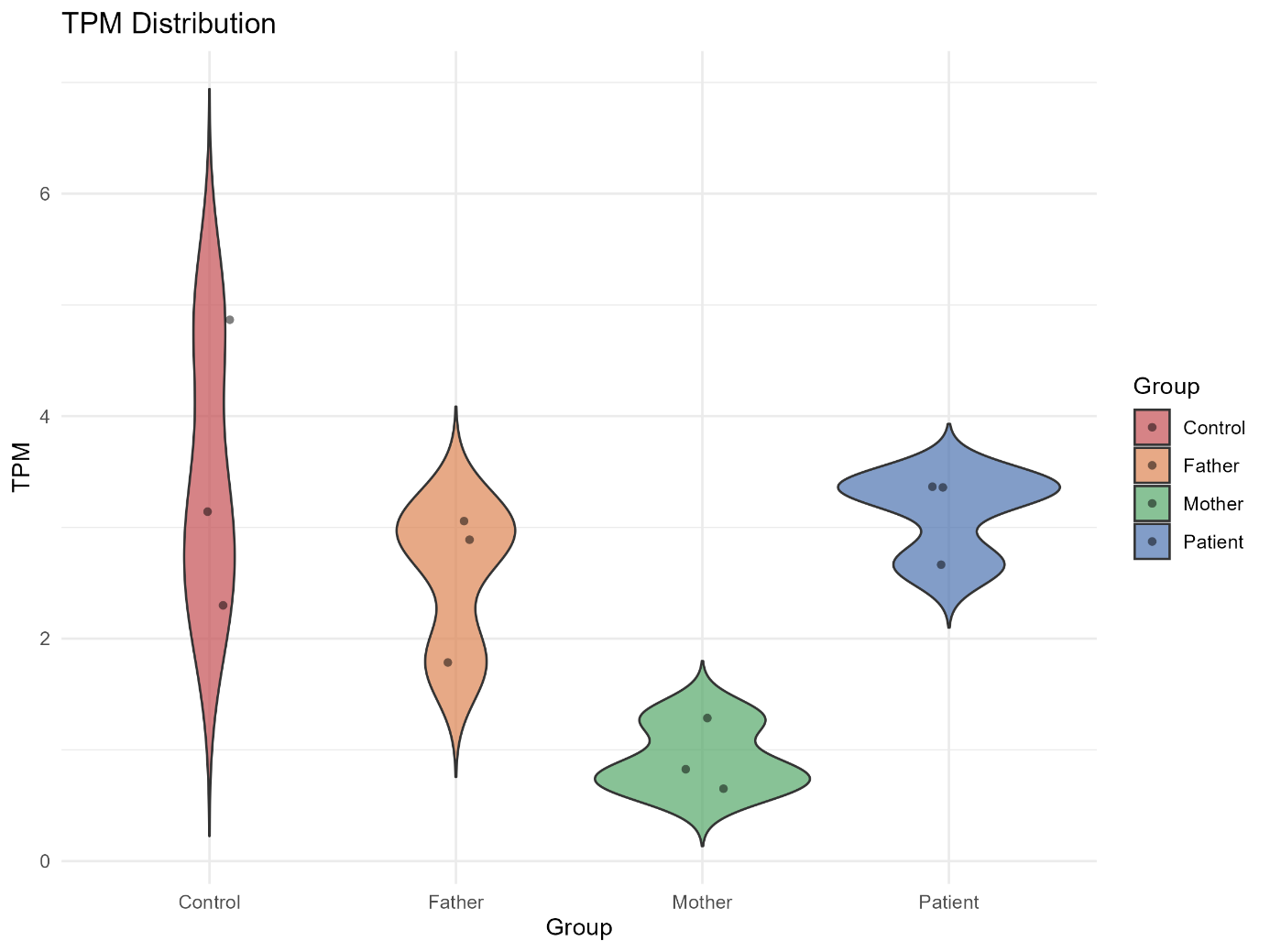


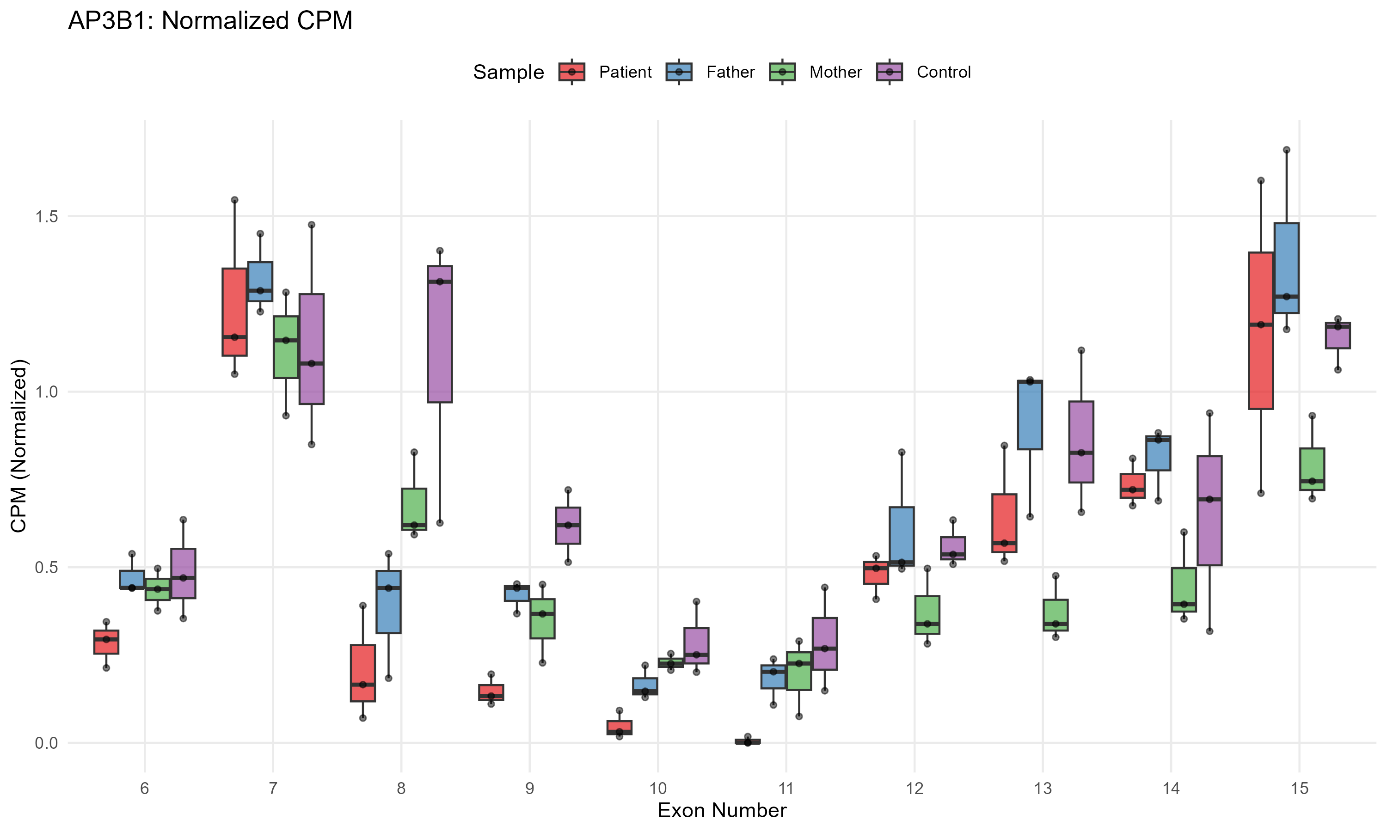


1. Violin plots of AP3B1 expression from RNA-seq. B) Exon-level counts per million of *AP3B1* derived from RNA-seq.

**Table S1**

| **patient** | **A** | **W** | **I** | **total** | **% of W** | **total A + W** | **% of A** | **Patient** |
| --- | --- | --- | --- | --- | --- | --- | --- | --- |
| KL_pt_1 | 29 | 13 | 4 | 46 | 0,309524 | 42 | 0,690476 | KL_pt |
| KL_pt_2 | 37 | 9 | 4 | 50 | 0,195652 | 46 | 0,804348 | KL_pt |
| KL_pt_3 | 38 | 11 | 3 | 52 | 0,22449 | 49 | 0,77551 | KL_pt |
| PL_par_1 | 24 | 28 | 6 | 58 | 0,538462 | 52 | 0,461538 | PL_par |
| PL_par_2 | 32 | 27 | 3 | 62 | 0,457627 | 59 | 0,542373 | PL_par |
| PL_par_3 | 20 | 18 | 8 | 46 | 0,473684 | 38 | 0,526316 | PL_par |
| YL_par_1 | 0 | 42 | 0 | 42 | 1 | 42 | 0 | YL_par |
| YL_par_2 | 0 | 31 | 0 | 31 | 1 | 31 | 0 | YL_par |
| YL_par_3 | 0 | 20 | 21 | 41 | 1 | 20 | 0 | YL_par |

mRNA-seq read counts covering the *AP3B1* locus exons 8-11. A: aberrant, missing these exons. W: wild type (containing at least two of exons 8-11). I: uninformative.
